# Experience does not change the importance of wind support for migratory route selection by a soaring bird

**DOI:** 10.1098/rsos.220746

**Published:** 2022-12-21

**Authors:** Hester Brønnvik, Kamran Safi, Wouter M. G. Vansteelant, Patrik Byholm, Elham Nourani

**Affiliations:** ^1^ Department of Migration, Max Planck Institute of Animal Behavior, Radolfzell 78315, Germany; ^2^ Department of Biology, University of Konstanz, Konstanz 78464, Germany; ^3^ Department of Wetland Ecology, Estación Biológica de Doñana, Seville 41092, Spain; ^4^ Theoretical and Computational Ecology, Institute for Biodiversity and Ecosystem Dynamics, University of Amsterdam, Amsterdam 1012 WX, The Netherlands; ^5^ Novia University of Applied Sciences, Ekenäs 10600, Finland; ^6^ Organismal and Evolutionary Biology, University of Helsinki, PO Box 65, 00100 Helsinki, Finland

**Keywords:** behavioural development, ontogeny, bird migration, step-selection function, *Pernis apivorus*

## Abstract

Migration is a complex behaviour that is costly in terms of time, energy and risk of mortality. Thermal soaring birds rely on airflow, specifically wind support and uplift, to offset their energetic costs of flight. Their migratory routes are a record of movement decisions to negotiate the atmospheric environment and achieve efficiency. We expected that, regardless of age, birds use wind support to select their routes. Because thermal soaring is a complex flight behaviour that young birds need to learn, we expected that, as individuals gain more experience, their movement decisions will also increasingly favour the best thermal uplift conditions. We quantified how route choice during autumn migration of young European honey buzzards (*Pernis apivorus*) was adjusted to wind support and uplift over up to 4 years of migration and compared this with the choices of adult birds. We found that wind support was important in all migrations. However, we did not find an increase in the use of thermal uplifts. This could be due to the species-specific learning period and/or an artefact of the spatio-temporal scale of our uplift proxies.

## 1. Introduction

Billions of animals migrate, engaging in a challenging behaviour during which environmental conditions affect fitness through survival and breeding success [1–3]. Migrating birds move through the air, which is in motion itself. The most important way for them to offset the energetic costs of movement is to ride airflow. Winds subsidize flight costs when birds move in the same direction as them (wind support), but increase costs of flight when flowing in the opposing direction (headwinds) or perpendicular to the birds (crosswinds) [4–6]. Birds can also take advantage of rising air (uplift) [7–9], whereas sinking air (subsidence) forces them to use powered flight to maintain altitude [10].

Soaring birds are among the most dependent on the dynamics of airflow due to their prohibitively high energetic costs of powered flight [11]. Under optimality theory [12,13], soaring birds will respond to the energetic costs and benefits of airflow by optimizing their travel within it. This is reflected in their small-scale movement decisions, which determine the energetic costs of larger scale movement behaviours, such as migration [14]. The migratory routes of these birds should be characterized by wind support [4,15] and uplift [11,16] to be energetically optimal. Yet not all individuals perform optimally. In many species, migratory routes vary in space and time within and between individuals [17,18], with first-year migrants performing less optimally than more experienced birds [19–22].

Whether these differences between juveniles and adults develop through a continuous process or a rapid acquisition of behaviour remains an important question in behavioural ecology [23–25]. Here we address this question by investigating how individuals' improvements in the use of airflow enable them to optimize their migration route choice with experience. We expect that juvenile soaring birds are able to use wind from an early age [26], as flying with wind support is not a cognitively complex task [27]. By contrast, soaring flight is complex, requiring the integration of cognitive processes (perception of the environment to locate thermals) and motor skills (adjusting flight speed and body angle within thermals) and is learned and perfected over time [7]. We therefore expect that younger migrants are not able to take advantage of thermals as efficiently as adults and that their movement decisions during migration reflect this.

We use a long-term dataset of GPS-tracked European honey buzzards (*Pernis apivorus*) to compare the influence of airflow on route choice during successive migrations by juvenile birds. Juvenile and adult honey buzzards differ in their migratory timing and routes. Adults depart the breeding grounds sooner [28] and may make long detours around water bodies [29]. Juveniles depart after adults, which leaves them unable to learn from informed conspecifics. They move with prevailing winds, apparently using compass direction and wind to determine their routes [26] and are more likely to perform long sea crossings [29].

We expect the adult behaviour to represent an attempt at optimality, and thus that as young birds gain experience their responses approach those of the adults. We expect that (i) wind support is an important determinant of route selection regardless of experience [15,26], and (ii) birds increasingly select their routes on the basis of uplift availability as they age [11]. Finally, wind support and uplift are not mutually exclusive and soaring birds can select routes by responding to one based on the condition of the other [30]. Inexperienced juveniles may be limited to using uplift when wind support is favourable [7,26]; we expect that (iii) whereas experienced birds maximize uplift regardless of wind support conditions, juveniles use uplift only when wind support is available.

## 2. Methods

### 2.1. Study system

We used existing data from a study of honey buzzards breeding in southern Finland (for details see Vansteelant *et al*. [26]). Between 2011 and 2014, buzzards were equipped with Argos or GPS transmitters; they were then tracked for up to 8 years. We analysed the routes taken on autumn migrations so that we could compare the first migration with subsequent journeys.

We analysed the autumn migrations of 23 fledglings from Finland to sub-Saharan Africa (electronic supplementary material, S1). In addition, three adults of unknown age transmitted four autumn migrations and two adults transmitted five autumn migrations. We analysed the fourth and fifth transmitted routes of these five adults because they are at least the fifth and sixth migrations (after at least the migration in juvenile plumage).

### 2.2. Step-selection functions

#### 2.2.1. Track processing

The transmitters had different sampling rates, ranging from 1 to 4 h. We subsampled the tracks of each individual to 1, 2, 3 or 4 h based on the median sampling rate of its transmitter so that time intervals between locations were consistent within each individual across years (electronic supplementary material, S2).

We analysed route selection using step-selection functions [31,32], which model movement as a series of discrete steps between consecutive locations, comparing conditions at locations that the birds used with those that were available but forgone.

We generated a stratified dataset (electronic supplementary material, S3) for the step-selection analysis. For each used step along the migratory route, we generated 100 available steps (electronic supplementary material, S4). We determined the end locations of available steps by randomly sampling from gamma distributions fitted to the step lengths and von Mises distributions fitted to the turn angles in the empirical data for each track (using the ‘amt’ package [33] in R [34]; electronic supplementary material, S5).

#### 2.2.2. Environmental data

We annotated all used and alternative locations using the Movebank Env-DATA service [35] to obtain data from the European Centre for Medium-Range Weather Forecasts (ECMWF) Global Atmospheric Reanalysis (ERA5). We considered two different proxies for uplift that are available through ECMWF: vertical velocity of pressure (Pa s^−1^), which quantifies vertical air movement, and planetary boundary layer height (m), which is dependent on rising air and therefore is higher where thermal uplift is strong. Both of these variables have been used by previous studies as proxies for uplift strength [36–39]. For each location, we retrieved east/west and north/south wind velocities (m s^−1^), vertical velocity and planetary boundary layer height. Because pressure is lower with increasing altitude, negative vertical velocity values indicate uplift [40]. All predictors are measured hourly at 0.25 degree (roughly 30 km) resolution and velocities are linearly interpolated at 925 mB pressure level (roughly 762 m.a.s.l.). We calculated wind support along each used and available step using the east/west and north/south wind velocities [41].

#### 2.2.3. Model fitting

We estimated step-selection functions using the integrated nested Laplace approximation (INLA) method of Bayesian inference (using the ‘INLA’ package [42] in R v. 4.0.2 [34]). We were interested in the importance of wind support, uplift and the interaction of the two to route selection and whether experience influenced the importance of these variables. We therefore included a three-way interaction term of uplift, wind support and migration year as our predictor. Migration year was included as a continuous variable. All adult birds of unknown age were assigned to migration year 5. We found a negative correlation between our two uplift proxies (vertical velocity and boundary layer height; *r* = −0.11; *p <* 0.05). Thus, we built separate models using the two uplift proxies, Model A with boundary layer height and Model B with vertical velocity as the proxy for uplift. To make the coefficients of our models comparable, we standardized the predictor variables across the whole dataset by calculating *z*-scores. In each model, we included individual ID as a random effect on the slopes. We set priors of N(0, 10^−4^) for fixed effects and set penalized complexity priors of PC(3, 0.05) to the precisions of the random slopes [43]. Finally, we assessed model fit using mean conditional predictive ordinates (CPO) and marginal likelihood (MLik). CPO is the probability of detecting a given observation if the model is fit excluding that observation, thus CPO detects outliers. High values of CPO are considered to show good predictive ability [44]. MLik is the joint probability of the data averaged over the prior. Smaller values of MLik are considered to show better fit [44]. We used CPO and MLik to compare the performance of the two models to decide which uplift proxy to use for interpreting the results.

## 3. Results

**Figure 1.**
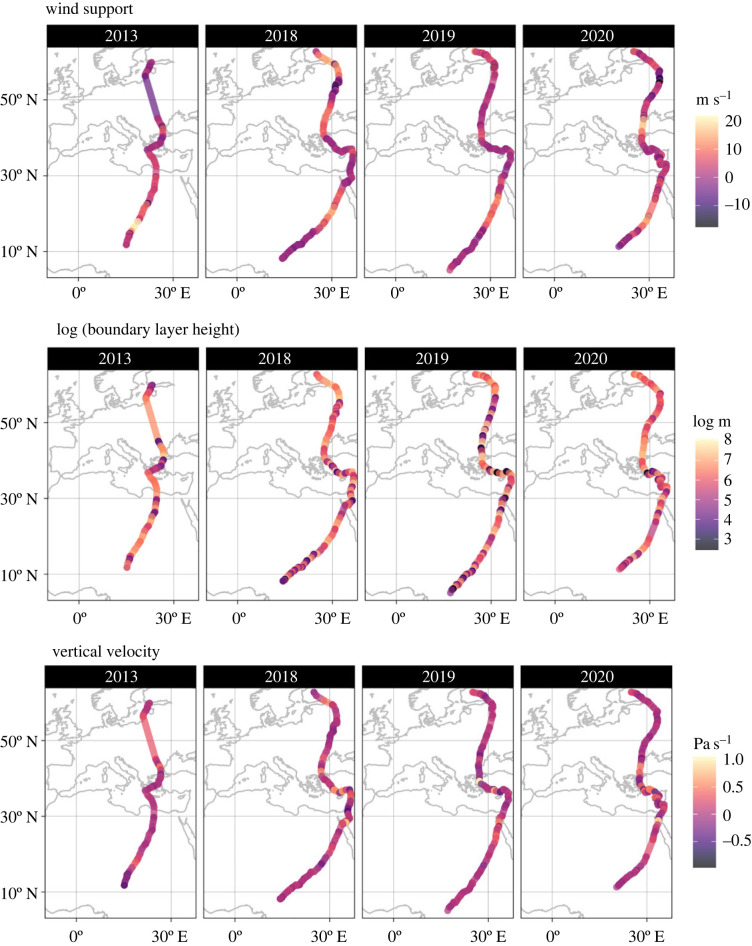
The tracks of a single individual tracked for four autumn migrations (columns left to right). Each track is labelled with the atmospheric conditions that were predictors in our models (rows).

Due to high juvenile mortality and/or tag failure, of our 23 first-time migrants only five transmitted a second autumn migration, four a third, and just two transmitted a fourth autumn migration. The atmospheric conditions along the routes of individuals that transmitted multiple autumn migrations did vary qualitatively over time (figure 1), but on average did not differ among migrations (electronic supplementary material, S6).

**Figure 2.**
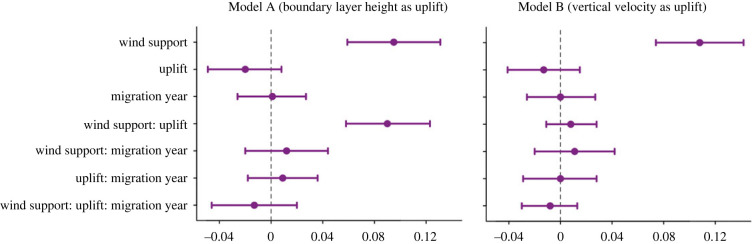
The importance of wind support, uplift and their interaction to route selection over migrations. Posterior means (centred and scaled) and 95% credible intervals for the fixed effects in the INLA models are shown.

The most important variable predicting route selection was wind support in both models (figure 2). In Model A (including boundary layer height), both wind support and the interaction of wind support with uplift were positive and important. In Model B (vertical velocity), only the effect of wind support was important. Migration year (1–5) was not an important predictor of route selection in either model. Models A and B performed equally (CPO = 0.97 for each, MLik = −38 517.73 and −38 534.20, respectively). We detected some non-significant individual variation in the wind support and uplift coefficients (electronic supplementary material, S7).

## 4. Discussion

We found that even young European honey buzzards exploit airflow when selecting their migratory routes and that there are no discernible differences between ages. We expected the importance of wind support for route selection of all ages [4,15,26], as flying with supportive wind is a cognitively cheap way of reducing flight costs [27]. Contrary to our expectation, there was no variation in responding to uplift among ages because neither uplift proxy was an important predictor of route selection for any age.

The role of uplift in route selection was inconsistent in our results. We used two different proxies for uplift—boundary layer height and the vertical velocity of pressure. Neither proxy was significant in our models. When we used boundary layer height, the interaction between uplift and wind support was shown to influence route selection positively. The importance of this interaction could be attributed to birds that are already travelling in good wind conditions selecting at the finer scale on good thermal conditions. However, when we used vertical velocity of pressure, the importance of the interaction disappeared. Based on these inconsistent results, we cannot make conclusions about the role of uplift in route selection for any age group.

Regardless of the proxy used, that we did not find any importance of uplift or an effect of age on route selection may be a matter of scale. The atmospheric data measured at roughly 30 km every hour might have failed to capture the uplift conditions that the birds select, which may be on a much finer scale [45] as they glide between thermal columns of varying diameters and strengths [46,47]. We examined selection at the scale of entire migratory routes, but birds make adjustments within and between thermals that we could not see, and capacity for these fine-scale adjustments may differ between ages [7,14]. Thus, weather models might not allow us to see the fine-scale improvements birds make after learning to soar and while they learn to soar efficiently. Proxies retrieved from weather models are not as reliable for measuring the proportion of a route spent soaring as data extracted from animals' movement are. Capturing this movement requires high temporal-resolution GPS data to show circling flight [48] and/or tri-axial accelerometry data to show flapping bouts [49]. These are not currently available for the honey buzzards.

To construct the developmental trajectories in selecting the optimal migratory route, we used a unique dataset that allowed us to compare the behaviour of the same juvenile individuals over 1 or more years of migration with individuals tagged as adults. The lack of variation in the behaviours among the different ages could indicate that the learning period is longer than 4–5 years. Because selecting optimal migratory routes may be cognitively demanding, requiring adequate perception of and responses to a changing environment, improvement in using airflow to optimize soaring flight and migratory performance may be slow and gradual [7,23,25]. In long-lived species such as the European honey buzzard, individuals may spend years acquiring and then refining their flight skills and migratory route selection [50].

The European honey buzzard could afford a long learning period because, as a facultative soaring species, its dependence on soaring flight is not strong. As a result, the cost of selecting routes on the basis of thermal availability might not be too high. This speculation could corroborate the findings of Sergio *et al*. that showed a shallow learning curve for the black kite [6,23], which is of a similar size. The birds tagged as adults in our study were individuals of unknown age and it may be that they were still immature and had not yet attained the optimal adult-like behaviour that we expected because they were still early on their learning curve. Our sample sizes (23 first-time migrants and five adults, electronic supplementary material, S2) might not have allowed us to capture the full learning curve. Thus, data collected for many individuals over extended time periods are required to understand how complex, cost-saving behaviours develop.

Route selection behaviour is more complex than simply reducing local energy expenditure, which was the basis of our expectations. Migratory decisions from departure time and travel speed to which routes to use are affected by many factors. Time is important among these as a currency governing migratory decisions along with energy; optimal migration is a compromise between minimizing time and maximizing energy gain [51,52]. In addition, factors such as predator avoidance [53], availability of roost sites [54] and food [55], and extreme conditions [1,56,57] contribute to migratory decisions in ways not considered here and that may differ between ages.

Migration is a complex behaviour that can be improved by experience [21,23,25]. We studied the ontogeny of migratory route selection in a long-lived, long-distance, soaring migrant in relation to airflow. We show that European honey buzzards use wind support to select migratory routes and that this does not change with experience. Our finding suggests that wind support is important for migration in all life stages of this species and we suspect that this may be the case in other facultative soaring species as well. This may have consequences for the longevity of the species in the face of shifting wind patterns driven by anthropogenic global warming.

## Data Availability

Data and R scripts used for step-selection analysis are available as electronic supplementary material [58].
